# Habitat Quality Affects Early Physiology and Subsequent Neuromotor Development of Juvenile Black-Capped Chickadees

**DOI:** 10.1371/journal.pone.0071852

**Published:** 2013-08-12

**Authors:** Thibault Grava, Graham D. Fairhurst, Marc T. Avey, Angelique Grava, James Bradley, Jillian L. Avis, Gary R. Bortolotti, Christopher B. Sturdy, Ken A. Otter

**Affiliations:** 1 Ecosystem Science and Management Program, University of Northern British Columbia, Prince George, British Columbia, Canada; 2 Department of Biology, University of Saskatchewan, Saskatchewan, Canada; 3 Departement of Psychology, University of Edmonton, Edmonton, Alberta, Canada; University of Lethbridge, Canada

## Abstract

In songbirds, the ability to learn and render the species-specific song is influenced by the development of both the song nuclei in the brain and the syrinx (bird's vocal apparatus) early in the bird's life. In black-capped chickadees (*Poecille atricapillus*), habitat quality is known to affect song structure, with birds in high-quality habitat (mature forest) having a higher song consistency than birds in low-quality habitat (young forest). Although this difference is suspected to stem from differences in development, the developmental status of juvenile birds in either habitat remains unexplored. In this study, we used ptilochronology and feather corticosterone to compare the conditional state of juvenile chickadees in young and mature forest during two distinct periods of song learning - the sensory phase, which occurs prior to settlement, and the sensorimotor phase, which occurs post-settlement. A sample of juvenile males was captured and euthanized several weeks prior to their first breeding season to compare the development of song center nuclei and syrinx in both habitats. The corticosterone levels of natally-grown feathers were greater among birds that settled in mature than young forests - as these feathers were grown pre-settlement, they reflect differences in physiology during the sensory phase. This difference in conditional state is reflected by differences in syrinx and song center nuclei development later during the sensorimotor phase - birds in young forest have smaller syrinx, and moderately-larger RA, than birds in mature forest. Those differences could be responsible for the difference in consistency in song structure observed across habitats. The difference in physiological state across habitats, combined with potential compounding effect of differences in winter resources between habitats, could influence the difference in syrinx and neural development seen in juvenile males during the early spring, and influence the male's ability to learn and render their species-specific song.

## Introduction

In songbirds, the learning, perception, and production of song are controlled by a set of interconnected brain nuclei known collectively as the ‘song system’ [1]–[4]. The song system is present in species that learn their song(s) through imitative learning of species-specific vocalizations [3], [4] during a sensitive period in early development [2], [5] that is divided into two sub-periods. Initially, there is a *sensory phase*, which is followed by the *sensorimotor phase* [1], [6]. In this second phase, the bird repeats the song initially memorized during the sensory phase, attempting to match this produced song to its internalized template [1]. Simultaneously, the brain nuclei of the song system undergo intensive developmental changes until the first breeding season, whereupon brain development is completed and the song crystallized [6]. These events are critical for normal song development; if there are perturbations to their development, birds do not properly produce their songs [7].

The *Developmental Stress Hypothesis* proposes that the condition of the birds during the period of song learning may affect the bird's neural development and consequently the song production quality [7]. In this study, Nowicki et al. found that the robust nucleus of the arcopallium (RA) and HVC (proper name), two brain nuclei of the song system [8], [9], were both significantly smaller in birds with nutritional deficit. Such condition-dependency may explain the acquisition of song types in birds with a vocal repertoire. For example, tarsus length correlated positively with song repertoire in blue tits (*Parus caeruleus*; [10]) and a significant relationship exists between feather growth and song repertoire in the great reed warbler (*Acrocephalus arundinaceus*; [11]). Food shortage - an important factor dictating a bird's condition - can impact the ability to learn and render songs during the nestling period (swamp sparrow *Melospiza georgiana*, [7]), the early post natal period (zebra finch *Taeniopygia guttata*, [12]), or when the birds are juveniles (European starlings *Sturnus vulgaris*, [13], [14]).

One environmental factor that may limit food availability, and thus impact the condition of birds, is habitat quality. The rate of feather regrowth (ptilochronology) can be used to infer individual condition under varying resources and habitat quality [15], [16]. In the Paridae (chickadees and titmice), wintering black-capped chickadees (*Poecile atricapillus*, hereafter, referred to as “chickadees”) in poor-quality habitat carry higher levels of furcular fat than do birds in high-quality habitat [17]; such high fat levels in other members of the Paridae family occur among birds that experience decreased or inconsistent access to food [18], [19].

Habitat quality is also known to influence condition-dependent behaviours in chickadees. Birds in young forest (low-quality habitat) have lower reproductive success [20], reduced territorial behaviour [21], and lower song output [22] than birds in mature forest (high-quality habitat). These condition-dependent behaviours appear to reflect low food access in poor-quality habitat, but have been measured primarily during the breeding season. In support of this hypothesis, Otter et al. [23] found subordinate female chickadees in young forest had higher food-solicitation calling rates than subordinate females in mature forests. Because food-solicitation calling rates are correlated with immediate hunger levels [23], the results suggest that birds in young forest had reduced access to food relative to birds in mature forest. Comparisons of somatic condition in chickadees during the breeding season indicate a greater disparity in provisioning rates among dominant and subordinate males occupying poor-quality versus good-quality habitat [24]. This suggests that resources may be limited and males in low somatic condition may reduce parental behaviour to compensate. Further, reduced song output at dawn that is seen across habitats [22] is alleviated by supplemental feeding in the early spring [25], suggesting a link between song output and the condition of the birds during the breeding season.

Although song output increases with supplementation of resources, other condition-dependent chorus signals do not, even though the immediate condition of the birds is increased. This is the case with the fidelity with which birds produce their songs; subordinate birds are less able than dominant birds to maintain relative frequency parameters between successive renditions of their songs [26]. Birds in high-quality habitat also show an increased ability to maintain consistent frequency structure between songs than do birds in poor-quality habitat [27]. Males assess rivals on this ability to accurately maintain internal note structure: males show lower response to perceived intruders with low consistency in song structure than they do to playbacks that maintain higher consistency in song structure [28].

These combined results suggest that the ability to maintain consistent song structure throughout the chorus is a condition-dependent signal in black-capped chickadees. However, food supplementation at the time of singing had no effect on this ability, suggesting that it does not reflect the short-term condition of the birds [27]. Rather, internal song structure consistency may reflect the condition of birds at the time of song learning, but no study has tried to estimate the early condition of chickadees and its implication on subsequent song structure. In chickadees, the sensory phase may occur as early as the nestling period [29], [30], which in our study population lasts approximately 18 days and is followed after fledging (late June to early July) by a period of dependency where the chicks are fed by their parents in the territory surrounding the nest for an additional two to three weeks. By mid-July to late August, juveniles disperse randomly and can travel as far as 10 km before settling into a flock with whom they will overwinter [31], [32]. Once settled, these birds will typically remain and attempt to breed in this settled habitat for the remainder of their lives [32]. It is during this post-settlement in the fall and early winter that the sensorimotor phase likely occurs. It is unknown, however, whether habitat affects the birds during the sensorimotor phase (i.e., post-dispersal) or whether the birds that settle in young and mature forest already differ prior to settlement.

To address these questions, we followed a cohort of chickadees settling in both young forests (poor-quality habitat) and mature forests (high-quality habitat) during their first winter. First in the fall, then subsequently during mid-winter, and again in early spring, birds were captured and feathers removed for analysis of growth rates and corticosterone (CORT) levels, because both can reflect responses to provisioning and environmental variation, including habitat [33]–[37]. We used ptilochronology and feather CORT levels as proxies of conditional state during two potentially important periods of song acquisition and development within the first year of male chickadees: the natal period, when it is thought that the birds might be memorizing song (*sensory phase*), and the post-dispersal/first winter period, when the song system (i.e., HVC, RA) is developing (*Sensory-motor phase*). A subset of males caught in early spring was then euthanized to assess neural and muscular development of the song nuclei and syrinx, respectively.

We investigated whether differences in conditional state exist in juvenile chickadees across habitat types during the two song learning phases. We predicted birds in young forest to be in lower condition, as measured by reduced feather growth and lower CORT levels, than birds in mature forest during at least one of these phases, and that this in turn would affect the development of the organs involved in song learning and song production, as measured by the volume of specific song nuclei (HVC and RA) and the weight of the syrinx. This study is the first to use feather-based measures to assess how physiological responses to ecological conditions during development influence the neuroethology of subsequent signaling behaviour.

## Materials and Methods

### Ethics statements

The feather collection described hereafter was carried out under permissions of the Canadian Wildlife Service bird banding office (CWS Permit 22806) and the UNBC Animal Care and Use Committee (Permit A2010.0120.003) in compliance with animal care and use guidelines for both Canada and the Association for the Study of Animal Behaviour. The euthanization of 20 juvenile males for brain and syrinx analysis was carried out under permissions of the UNBC Animal Care and Use Committee (Permit A2009 0119 013).

### Study site

The study took place in Prince George, British Columbia, Canada (53°55″01′N 122°44″58′W) on three plots of mature forest, at least 850 m apart, and four plots of young forest at least 3.7 km apart. Plots of both types were intermixed throughout the study region (which covered 16.35 km from N to S and 13.90 km from E to W, centered on the city), and each study plot consisted of forest of homogenous age.

The three mature sites were characteristic of the mixed woodlands of the sub-boreal spruce sub-zone in Northern British Columbia. Further, all mature sites were similar in age structure, with no commercial logging for more than 80 years. Dominant deciduous species in these sites are trembling aspen (*Populus tremuloides*), paper birch (*Betula papyrifera*), and black cottonwood (*Populus balsamifera ssp trichocarpa*). Subalpine fir (*Abies lasiocarpa*), lodgepole pine (*Pinus contorta*) and hybrid spruce (*Picea glauca* x *Picea engelmannii*) form the predominant conifer species in these mixed forests, but tend to be less abundant than deciduous species. All these sites were characterized by an average trunk diameter of 25 cm and a canopy at approximately 25 m.

The four young sites were forests that have undergone near-complete clearing in the past 30years. All young sites had characteristic flora of a young, regenerating sub-boreal forest. Dominant deciduous tree species and conifer species were similar to those described above for the mature sites, but early successional species, such as lodgepole pine, dominated the coniferous component. These sites were characterized by an average trunk diameter of 10 cm and a canopy at approximately 8 m.

### Song learning in black-capped chickadees

In wild populations of black-capped chickadees, juveniles start producing whistled approximations of song at approximately day 20 post-hatch ([29], confirmed by personal observation in the field), within days of fledging. We feel confident that the sensory phase begins prior to this date when the birds are still fed by their parents (i.e., from hatching to fledging) and that the sensorimotor phase begins once chickadees disperse [30]. This sensorimotor phase lasts until the birds enter their first breeding season [29], [30], [6].

During the entire song learning period the song system develops in parallel with the acquisition of adult song (for review see [2]). As a songbird, the template for the tutor's song in the black-capped chickadee is thought to be encoded in the neural substrate of NCM (caudomedial nidopallium) and CMM (caudomedial mesopallium; [1]). During development, the anterior forebrain pathway (AFP) is involved in the acquisition of song as well as auditory-vocal feedback processing [38]. The AFP connects the brain nuclei for learning of song including Area X (of the striatum), HVC, LMAN (lateral magnocellular nucleus of the anterior nidopallium), and RA [2]. The posterior pathway or song motor pathway (SMP) controls the production of song and includes HVC and RA which innervate nXIIts (tracheosyringeal portion of the nucleus hypoglossus) that controls the syrinx [38]. Normal development of the song system is crucial during the sensitive period for normal development of song [7]; [27]; [28].

### Feather collection

We trapped birds in three sessions during the period of Aug 2009 to April 2010. The first session was conducted between 28 August and 6 September 2009 inclusively (Fall), the second was conducted between 11 November 2009 and 21 January 2010 inclusively (Winter), and the third between 23 March and 12 April 2010 inclusively (Spring). We used a variety of techniques to attract the birds into mistnets, including playback of *chick-a-dee* calls (recorded in the context of either food-finding or mobbing), presentation of feeders filled with sunflower seeds, and presentation of a stuffed chickadee (intruder) or saw-whet owl (predator). In most circumstances, combinations of these stimuli were used to trap birds and all techniques were used the same way in both habitat.

Only birds that hatched during the preceding spring (identified by shape and wear of outer rectrices [39]) were sampled for this study. To make sure no dispersing birds entered the analysis, only birds that we know were established for at least a full season or were established at the onset of their first breeding season in a particular habitat entered the analysis. We plucked the two outer tail feathers at the beginning of the first fall (August) of 120 birds (60 in each habitat), then color-banded them and released them; these “natal” feathers reflect those grown in the nest and during early post-fledging in juvenile chickadees [32]. This plucking induced feather regrowth during the fall, which corresponds to the beginning of the sensorimotor phase. In the winter period (December – January), we recaptured 26 from the fall sample (14 in young forest and 12 in mature forest), as well as an additional 30 new (i.e., unbanded) birds. For recaptured birds, we plucked the induced feathers for assessment of ptilochronology and feather CORT during the first fall period (see below). For new birds we plucked feathers so as to induce regrowth in the winter. Finally, for spring sampling (April), we returned to the same capture locations and recaptured six birds previously-banded in the fall (out of the 30 previously banded birds; four in young forest and two in mature forest) and 16 birds not previously captured from which we plucked the natal feathers (nine in young forest and seven in mature forest). In total, the natal feathers from 48 birds (26+6+16) gave us data from the sensory phase, the feathers from the 32 recaptured birds (26+6) gave us data from the sensorimotor phase. For each bird, we measured growth bars on one tail feather using ptilochronology (see below), and used the other feather for CORT analysis. All feathers were mature (i.e., fully-grown) when collected.

### Ptilochronology

Ptilochronology is the study of daily growth using feathers [40]. Tail feathers record evidence of their daily growth in visible “bars” that can be measured, similar to growth rings on tree. The width of these Daily Growth Bars (DGBs) correlates with food availability, and thus ptilochronology can be used as an index of relative condition of individuals under different ecological conditions [34].

The methods for measuring the width of DGB on the feathers were taken from Grubb [40]. Briefly, the feathers were placed on a card and pinned at their extremities. We measured the total length of each feather and calculated and marked the location of the ^2^/_3_ position along the length of the feather from the proximal end. We then inserted a pin perpendicularly through the feather at the distal edge of each DGB. We then determined the average DGB width using the mean length of 10 DGBs centered on the ^2^/_3_ point with four additional DGBs proximal to and five distal to the central DGB. This measure gave us the average DGB width of the natal feathers that grew during the first few weeks after the birds fledge, and of the induced feathers of the birds we recaptured.

### CORT analysis

Feather CORT assays followed Bortolotti [41]. Briefly, we extracted CORT from feathers using a methanol-based technique. The length of the feather was measured, the calamus was removed and discarded, and then the sample was cut into pieces <5 mm^2^ with scissors. We then added 10 mL of methanol (HPLC grade; Fisher Scientific, Fairlawn, New Jersey, USA) and placed the samples in a sonicating water bath at room temperature for 30 min, followed by incubation at 50°C overnight in a shaking water bath. To separate the methanol from the feather material, we used a vacuum filtration system that included a plug of synthetic polyester fibre in a filtration funnel. The methanol extract was placed in a 50°C water bath and subsequently evaporated in a fume hood. Extract residues were reconstituted in a small volume of phosphate buffer saline (PBS; 0.05 M, pH 7.6) and frozen at −20°C until analyzed by radioimmunoassay (RIA). We assessed the recovery of the methanol extraction by including feather samples spiked with a small amount (approximately 5000 CPM) of ^3^H-corticosterone in the extraction. Samples were extracted in two batches, and 92% and 96% of the radioactivity was recoverable in the reconstituted samples, respectively. Final values were adjusted by recoveries. For more information about validation, see Supplementary Appendix S1 available in online version of Bortolotti [41].

Feather CORT levels were determined by RIA as in previous studies [40]–[43], [34]. Measurements were performed on 100 µL of reconstituted methanol extracts that had been reconstituted in PBS and were duplicated. All samples and standards were incubated with 100 µL of tritiated CORT (∼5000 CPM) at room temperature for 18 hours. Dextran-coated charcoal was used to separate bound from free hormone. Of the known hormones and CORT metabolites in feather extracts ([41], [44]), the antiserum we used (Sigma-Aldrich C8784, Oakville, Canada, lot # 090M4752) cross-reacts with cortisol (4.5%) and testosterone (7.9%), but evidence suggests that this antiserum is reacting with unmodified CORT (see Supplemental Appendix in [41]). Antiserum was diluted to obtain specific binding of 25–30%. Samples were measured in two assays and we assessed assay variability and precision by including, in each assay, three additional CORT samples of identical concentration. Based on these internal standards, the Samples were measured in two assays with an intra-assay coefficient of variation of 8.2% and, an inter-assay coefficient of variation of 9.6%. The mean (±SD) limit of detection (ED80), calculated as the hormone value with a relative binding (% B/B0) of 80% (ED80) was 10.63±0.26 pg CORT per assay tube, though all samples were above this limit. Data values are expressed as pg CORT per mm of feather, which gives a valid estimate of CORT per unit time of feather growth (for validation see Supplemental Appendix S1 in [41], [45], [46]).

### Brain and syrinx analysis

#### Histology

During spring sampling, and after winter flocks broke up in early April (after song crystallized), 20 second-year males hatched the previous summer were euthanized for brain and syrinx histology. Ten males from either of the two habitat classes were sampled and euthanized within one hour of capture. Sex was confirmed via inspection of gonads following euthanasia. Birds were euthanized with 0.03 ml of 100 mg/ml ketamine and 20 mg/ml xylazine given intramuscularly (1∶1) and then transcardially perfused with heparanized 0.1 M phosphate buffered saline (PBS) followed by 4% paraformaldehyde. All efforts were made to minimize suffering between capture and euthanization. Following perfusion, the brain and syrinx were removed and placed in 4% paraformaldehyde. Measurements and tissue preparation was carried out blind to the habitat condition of the specimens and specimens were selected in a random order. After one week, the brain and syrinx were weighed three times each and the median value was recorded. The brain was then placed in 30% sucrose in PBS for approximately 24 h until saturated. The brains were subsequently snap-frozen in isopentane at −80°C and stored at −80°C. Brains were then warmed to −20°C and the entire brain was sectioned coronally at 40 µm using a cryostat and directly mounted on gelatin-embedded slides in four series. The first series of mounted sections, every fourth section, were then stained with Thionin (0.25%) and coverslipped.

#### Brain Morphometry

To calculate total brain volume we used methods similar to previous research [47], [48]. First, each slide was scanned into ImageJ, using an HP4470c ScanJet. The total area of the brain as well as the total areas of the brain nuclei for HVC and RA in every section in which they appeared were traced using a WACOM tablet. The total volume of the brain, HVC and RA were calculated by multiplying the given surface area by the distance between measurements [46]. Overall brain size was accounted for by dividing the volumes of HVC and RA by total brain volume. All imaging and tracing was done by a research assistant blind to the habitat condition of the specimens.

### Statistical analysis

To determine whether CORT levels should be controlled for when analyzing DGBs, and vice and versa, we analyzed the correlations between those factors during the two learning phases.

#### Condition during sensory phase

We used General Linear Models (GLM; STATISTICA version 8.0 StatSoft, Inc) to compare the levels of CORT in the natal feathers (dependent variables; n = 46) between habitats in which the birds were sampled (categorical variables, mature n = 21, young n = 25) controlling for period of feather collection (categorical variables, fall n = 24, winter n = 22), sex (categorical variables; female n = 11, male n = 19) and width of DGBs in the same natal feathers (continuous variables, n = 46). We used an additional GLM to compare the natal DGBs (n = 46) between habitats in which the birds were sampled (mature n = 21, young n = 25), controlling for period of feather collection (fall n = 24, winter n = 22) as well as for the CORT in the same natal feathers (n = 46).

#### Condition during sensorimotor phase

We used a GLM to compare the levels of CORT in induced feathers (dependent variable, n = 30) between habitats in which the birds were sampled (categorical variable; mature n = 14, young n = 16), controlling for the period in which the feather grew (categorical variables; fall n = 24, winter n = 6), sex (categorical variables; female n = 11, male n = 19) as well as for DGBs of the same feathers (continuous variable; n = 30). To control for previous condition, we additionally included as covariates the DGB and CORT values (n = 30) from the natal feathers previously sampled from the same birds.

We used a GLM to compare the levels of DGB in the induced feathers (dependent variable; n = 30) between habitats in which the birds were sampled (categorical variable; mature n = 14, young  = 16), controlling for the period in which the feather grew (categorical variables; fall n = 24, winter n = 6) as well as for the CORT in the same feathers (continuous variables; n = 30). To control for previous condition, we additionally included as covariates the DGB and CORT values (n = 30) from the natal feathers previously sampled from the same birds entering this analysis.

#### Brain and syrinx measurements after song crystallization

The correlations between anatomical and physiological parameters were analyzed using regression analysis. However, our sample size of birds euthanized per habitat was limited (by ethical considerations) to ten. We used an ANOVA to compare syrinx weight between habitats because the power of that test (63%) was robust. However, the power of all comparisons of song nuclei across habitat was below 14%. We therefore used Cohen's d to compare the effect size of differences in brain measurements between forests (d = 0.1 represents a small effect, d = 0.3 an intermediate effect, and d = 0.5 a large effect; [49]). We used a regression analysis to detect any correlation between syrinx weight, HVC and RA volume with CORT and DGBs from natal feathers of the same birds.

## Results

### Condition during sensory phase

CORT levels in natally-grown feathers were negatively correlated with DGBs (regression analysis, R^2^ = 0.11, N = 46, p = 0.02) and therefore were controlled for in the subsequent analysis.

**Figure 1:**
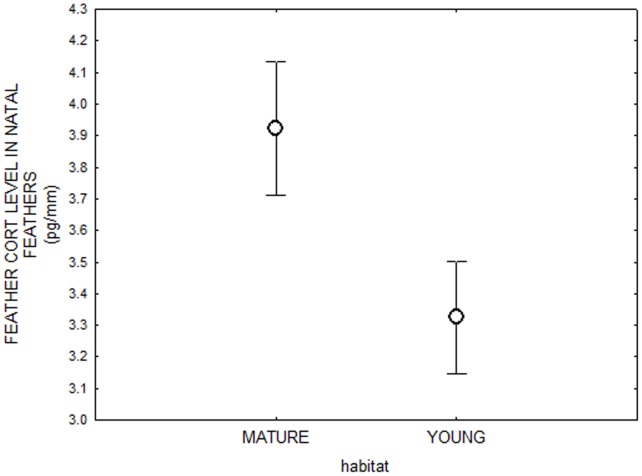
Comparing CORT levels in juvenile birds from young and mature forest during the sensory phase. CORT levels (mean ±SEM) in natal feathers are higher in birds that settled in mature forests compared to birds settling in young forests (General linear model, F_1,37_ = 4.71, N = 46).

**Figure 2:**
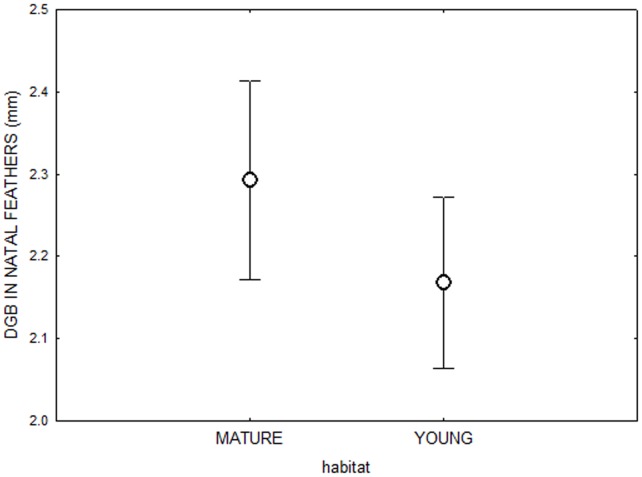
Comparing DGBs of juvenile birds from young and mature forest during the sensory phase. Mean (±SEM) growth bar widths (DGB) in the natal feathers of birds that settled in either mature or young forests (General linear model, (F_1,37_ = 0.35, N = 46, p = 0.55. Dots represent mean and whiskers error bars.

Natal feather CORT was significantly higher in birds that settled in mature forests compared to birds settling in young forests (General linear model, F_1,37_ = 4.71, N = 46, p = 0.03; Figure 1). However, when controlling for CORT in natal feathers, there was no significant difference in DGB in the original feathers of birds that settled in either mature or young forests (F_1,37_ = 0.57, N = 46, p = 0.45; Figure 2).

### Condition during sensorimotor phase

CORT levels in induced feathers were negatively correlated with DGBs (regression analysis, R^2^ = 0.31, N = 46, p = 0.001) and therefore were controlled for in the subsequent analysis.

We found no statistically-significant differences in CORT levels of induced feathers from birds settling in the two forest ages, (F_1,21_ = 2.13, N = 30, p = 0.16). We also found no significant difference between DGBs in induced feathers from young vs. mature forest (F_F1,21_ = 0.31, N = 30, p = 0.58).

### Brain and syrinx measurement after song crystallization

**Figure 3:**
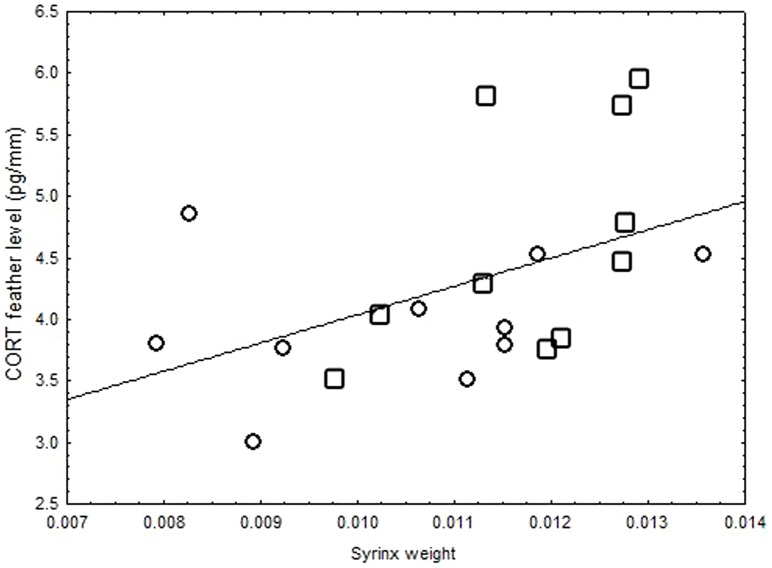
Correlation between CORT levels during sensory phase and syrinx weight. Syrinx weight of black-capped chickadee in their first spring is correlated with CORT levels of their natal feathers (regression analysis, R^2^ = 0.1, N = 20, p = 0.03).Circles represent birds from young forest; squares represent birds from mature forest.

Syrinx weight in the first spring correlated significantly with CORT levels of the natal feathers (R^2^ = 0.1, N = 20, p = 0.03; Figure 3) but not with DGB in these feathers (R^2^ = 0.003, N = 20, p = 0.79). Neither HVC nor RA volumes were correlated with CORT levels (R^2^ = 0.08, N = 20, p = 0.23; R^2^ = 0.04, p = 0.38; respectively) nor DGB in natal feathers (R^2^ = 0.003, N = 20, p = 0.79; R^2^<0.0001, p = 0.98; respectively). As several of these males were only caught in the spring sampling period, there was insufficient data to compare syrinx or brain measures to CORT or DGB of re-grown feathers.

**Figure 4:**
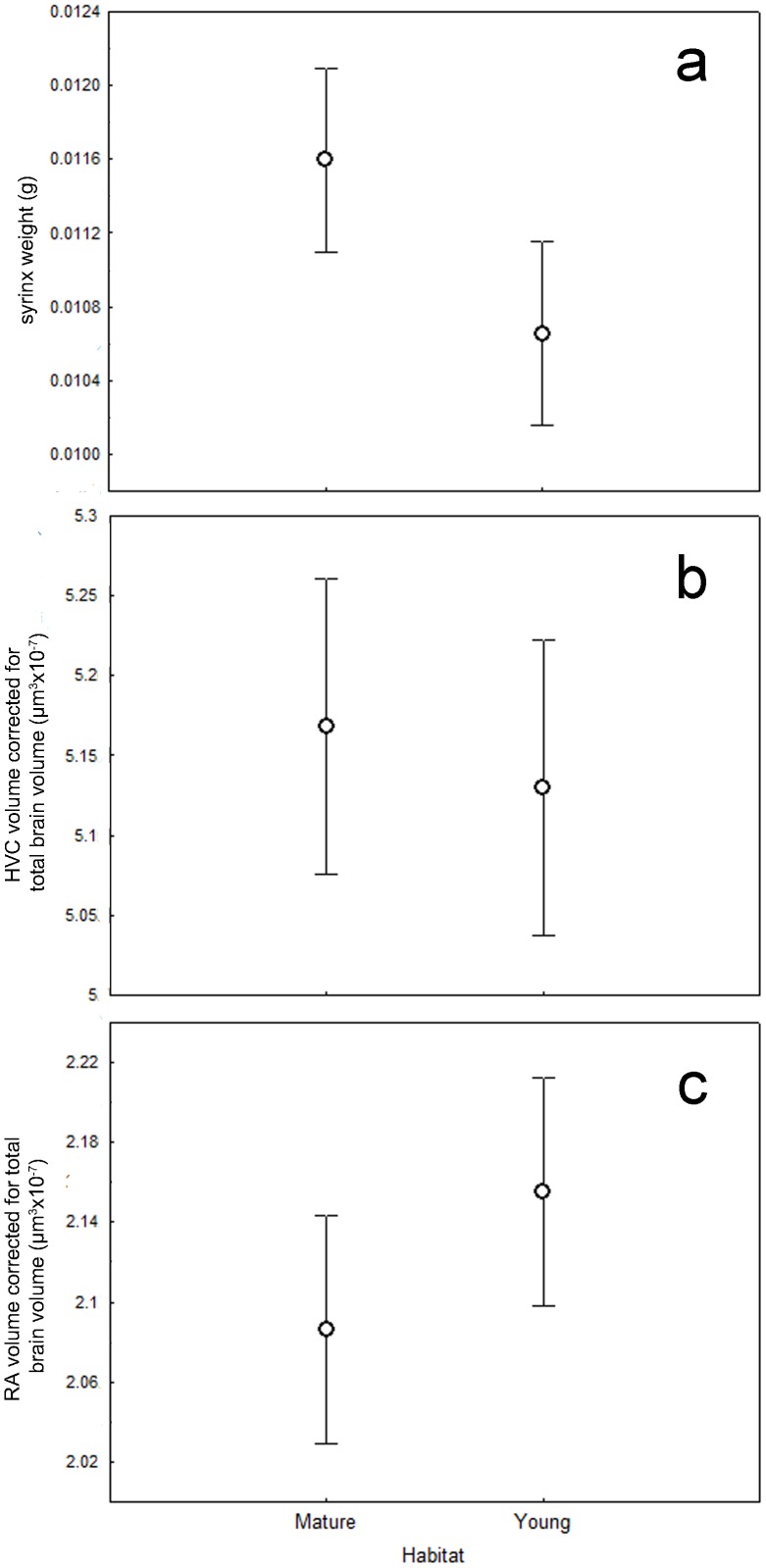
Comparing the development of syrinx, RA and HVC across habitat. (a) Birds from young forests have a lighter syrinx than birds in mature forest (F_1,17_ = 5.86, N = 20, p = 0.02). (b) An intermediate effect (based on power analysis) was found between RA volume in young vs. mature forests; birds in young forests tend to have a bigger RA volume than birds in mature forest (F_1,17_ = 0.81, N = 20, p = 0.38; d = 0.38). (c) HVC volume did not differ significantly across habitats (F_1,17_<0.01, N = 20, p = 0.99; d = 0.13). Values presented are Means ±SE.

Birds from young forests had a significantly-lighter syrinx than birds in mature forest (F_1,17_ = 5.86, N = 20, p = 0.02; Figure 4a). Although not statistically significant, power analysis suggested an intermediate effect existed between RA volume in young vs. mature forests: birds in young forests tended to have a bigger RA volume than birds in mature forest (F_1,17_ = 0.81, N = 20, p = 0.38, d = 0.38; Figure 4c). HVC volume did not differ across habitat (F_1,17_<0.01, N = 20, p = 0.99, d = 0.13; Figure 4b); it was neither statistically significant, or the effect size indicative of more than slight differences between groups.

## Discussion

Our study compared the conditional state of juvenile chickadees settling in young versus mature habitat during the two phases composing the song learning period, and determined whether habitat type affected the development of organs allowing song learning – song nuclei (HVC and RA) and the syrinx. We found that CORT from natal feathers predicted the type of habitat in which the birds ultimately settled during the fall. In the subsequent spring, at the onset of their first breeding season, birds that had settled in young forest had significantly lighter syrinxes than birds that settled in mature forest. Importantly, syrinx weight was positively correlated with natal feather CORT.

### CORT difference across habitat

Our feather CORT results suggest that birds that ultimately settled in mature and young forests differed physiologically during the period of natal feather growth. All previous studies of chickadees settling in similar habitat types in the region indicate that birds in young forest likely encounter higher levels of resource limitation [20]–[23], [25], [50], and the influence of forest age on CORT likely acts through food supply [29]. Food restrictions and poor quality diet typically result in an increase of plasma CORT in nestling birds [50]–[54]. With regards to feather CORT, diminished provisioning rates in Cory's shearwater (*Calonectris diomedea*) chicks were likely responsible for lower feather CORT, suggesting a positive relationship between energetic condition and feather CORT [35], a result also found using plasma CORT levels [55]. Such a relationship may not be likely in our study because the life-history strategy of chickadees differs significantly from that of shearwaters. However, plasma CORT levels in altricial nestlings do not always increase in response to nutritional stressors [56] and can even decrease during moult [57], leaving habitat differences in food supply a potential explanation of our results. It is additionally possible that nestling feather CORT reflected energy management. For example, increased variability in nest box microclimate was positively related to nestling feather CORT in tree swallows (*Tachycineta bicolor*), but variation in feather CORT was not large and likely did not indicate stress per se [58]. Like tree swallows, chickadees are cavity nesters, and our observed differences in feather CORT were small, so our results may reflect subtle energetic adjustments to conditions experienced as nestlings, or possibly developmental effects [58]. Feather CORT values can reflect changes in plasma CORT over the entire period of feather growth [41], [59] which, in our study, occurred in the nest. However, we did not measure plasma CORT during feather growth so were not able to determine what specific differences in CORT physiology (e.g., baseline or stress-induced CORT), stressors, or their timing account for the observed variation in feather CORT. Regardless, small but significant differences in feather CORT predicted subsequent habitat selection, which is intriguing, especially considering these differences did not persist through winter (see below). The observed link between CORT and habitat is surely multi-factorial and needs to be addressed with additional longitudinal studies. In any case, it seems that the establishment of the birds into good and poor-quality habitat may be linked to early-life environment and natal physiology.

### Despotic distribution

Our results suggest that the physiology of black-capped chickadee nestlings may predict whether the birds will establish in good or poor-quality habitat. A similar study showed that prior to fall settlement chickadee DGBs did not differ across habitat [17], and the authors concluded that there was no evidence for differential settlement (at least among males) based on individual condition. Our ptilochronology results echo this conclusion. However, by using feather CORT as a measure of responses to environmental conditions in the natal area, we were able to detect a difference between juvenile birds settling in different-aged forests. If feather CORT is an indicator of chickadee condition, as suggested by our results, a despotic distribution may occur when juveniles disperse: the birds in better condition tend to establish in higher-quality habitat, while birds in lower condition are pushed towards sub-optimal habitat [60].

### Condition and development during song learning

We found that birds settling in young forest had lower CORT levels during the sensory phase of song learning than birds settling in mature forest; during the sensorimotor phase, CORT levels did not differ between habitat types. However, our study of the CORT levels in feathers during the sensorimotor phase suffers from a smaller sample size compared to the study of the sensory phase, and this could explain the lack of consistency between those results. The difference between sensory phase versus sensorimotor phase CORT levels could not be explained by birds in young forest benefitting from the lack of competition in this habitat since no difference in chickadee's population density exists across habitat (TG, AG, KAO, personal observations). It is possible that, regardless of habitat type, winter conditions are challenging for hatch-year birds. Alternatively, if conditions such as weather and food supply were particularly favorable in both habitat types during winter feather growth, nestlings may be expected to have similar CORT levels. To shed light on these hypotheses, future studies should identify additional environmental, behavioral, and physiological variables that differ among individuals within the two habitat types.

The sensory phase occurs when birds are still nestlings and is extremely important for song development. Birds in poor condition during this phase have a lower ability to learn their species-specific songs because of a reduced development of the organs involved in song learning [7]. We found a physiological difference between birds that eventually settle in young forest compared to those in mature forest. The correlation between CORT levels in natal feathers and the syringeal mass in juvenile males entering their first breeding season suggests a connection between responses to natal conditions and song learning. Syrinx mass was also smaller among our young-forest birds than the mature-forest birds. Because a smaller syrinx is associated with lower imitation of a tutor song [61], our results may explain why motor production of songs among birds from young-forest tends to be less consistent than those of birds from mature-forest [27]. The fact that syrinx weight is correlated with natal feather CORT suggests a link between the conditional state of chickadees during the sensory phase and later development of the neuro-motor pathways responsible for song learning.

Although our sample size may have hampered our statistical analysis on potential differences between song center volumes across habitat, our results still suggest that differences may exist across habitats. Birds in young forests tend to have a larger RA volume than those in mature forests. Other studies suggest that HVC is affected more by early condition than RA. Zebra finches that have undergone developmental stress during song learning (through nutritional and hormonal stress, or parasitic infections) have smaller HVC region, while RA volume or brain size was not affected [62]–[64]. Our results show that even though birds in young forest tend to have a larger RA volume, they still have a reduced ability to maintain internal song structure [27].

Our study may provide a mechanistic explanation of why adults birds in these same habitats differ in the ability to produce songs with consistent frequency ratios [27], and the ability of maintaining these components of song structure appear to positively influence male perception of intruder condition (unpublished data). That we found no differences in conditional state in birds in both habitats during the sensorimotor phase might reflect a trade-off where birds with limited energy reserves invest first in somatic maintenance (body condition) and less in the development of sexually-selected traits [55]. This may explain why we observe little difference in the condition of birds between good and poor-quality habitats, but major differences in the expression of sexually-selected signals.

### Conclusion

Our study substantiates a link between habitat quality of natal areas, individual physiology, and development in a generalist bird. We have shown that habitat quality during early life has a profound effect on development and consequently on the ability to learn species-specific song. This can have very important effects on the bird's local population, particularly if low-quality habitat continues to expand, increasing the impact on developing birds' song learning, song production, and ultimately, having negatively impacting fitness.
